# Liraglutide, a Glucagon-Like Peptide-1 Analog, Increased Insulin Sensitivity Assessed by Hyperinsulinemic-Euglycemic Clamp Examination in Patients with Uncontrolled Type 2 Diabetes Mellitus

**DOI:** 10.1155/2015/706416

**Published:** 2015-04-02

**Authors:** Hideaki Jinnouchi, Seigo Sugiyama, Akira Yoshida, Kunio Hieshima, Noboru Kurinami, Tomoko Suzuki, Fumio Miyamoto, Keizo Kajiwara, Kunihiko Matsui, Tomio Jinnouchi

**Affiliations:** ^1^Diabetes Care Center, Jinnouchi Hospital, 6-2-3 Kuhonji, Chuo-ku, Kumamoto 862-0976, Japan; ^2^Division of Preventive Cardiology, Department of Cardiovascular Medicine, Kumamoto University Hospital, 1-1-1, Honjo, Chuo-ku, Kumamoto 860-8556, Japan; ^3^Diabetes Care Center, Cardiovascular Division, Jinnouchi Hospital, 6-2-3 Kuhonji, Chuo-ku, Kumamoto 862-0976, Japan; ^4^Department of Cardiovascular Medicine, Faculty of Life Sciences, Graduate School of Medical Science, Kumamoto University, 1-1-1, Honjo, Chuo-ku, Kumamoto 860-8556, Japan; ^5^Department of Community Medicine, Kumamoto University Hospital, 1-1-1, Honjo, Chuo-ku, Kumamoto 860-8556, Japan

## Abstract

*Aims*. Glucagon-like peptide-1 (GLP-1) analog promotes insulin secretion by acting on pancreatic *β*-cells. This antihyperglycemic treatment for type 2 diabetes mellitus (DM) has attracted increased clinical attention not only for its antihyperglycemic action but also for its potential extrapancreatic effects. We investigated whether liraglutide, a GLP-1 analog, could enhance insulin sensitivity as assessed by the hyperinsulinemic-euglycemic clamp in type 2 DM patients. *Materials*. We prospectively enrolled 31 uncontrolled type 2 DM patients who were hospitalized and equally managed by guided diet- and exercise-therapies and then introduced to either liraglutide- or intensive insulin-therapy for 4 weeks. Insulin sensitivity was assessed by the glucose infusion rate (GIR) using hyperinsulinemic-euglycemic clamp before and after the therapies. *Results*. Values of HbA1c, postprandial plasma glucose, and body mass index (BMI) were significantly decreased by hospitalized intensive insulin-therapy or liraglutide-therapy. GIR was significantly increased by liraglutide-therapy but not by insulin-therapy, indicating that liraglutide-therapy significantly enhanced insulin sensitivity. BMI decreased during liraglutide-therapy but was not significantly correlated with changes in GIR. Multivariate logistic regression analysis demonstrated that liraglutide-therapy significantly correlated with increased insulin sensitivity in uncontrolled DM patients. *Conclusions*. Liraglutide may exhibit favorable effects on diabetes control for type 2 DM patients by increasing insulin sensitivity as an extrapancreatic action. Clinical trial registration Unique Identifier is UMIN000015201.

## 1. Introduction

The number of patients with type 2 diabetes mellitus (DM) has rapidly increased globally. New pharmacological agents have been developed for the management of hyperglycemia and type 2 DM [1]. Incretin-related medications have attracted great clinical attention for the treatment of DM [2, 3]. It has been reported that the GLP-1 analog, which is an incretin-related medication, shows antihyperglycemic action by enhancing insulin secretion from pancreatic *β*-cells, but the unique effects of this treatment are pleiotropically expected as extrapancreatic action including appetite loss, body weight loss, and delayed gastric emptying [4]. A detailed examination of the antihyperglycemic action and mechanisms of the GLP-1 analog is warranted in the present clinical situation. The future applications of the GLP-1 analog are important not only in diabetes but also in obesity resulting in the recent clinical interest in the extrapancreatic action of the GLP-1 analog [5, 6].

Investigations of impaired insulin sensitivity are mainly involved in the pathogenesis of type 2 DM. Several animal studies have demonstrated that GLP-1 and GLP-1 analog exhibit the ability of improving insulin sensitivity [7]. A GLP-1 analog has been shown to activate AMP-activated protein kinase (AMPK) in* in vitro* experiments [8]. The AMPK activation in the extrapancreatic organs, including the liver and muscle, can enhance insulin sensitivity [9]. An early clinical study with a small number of DM patients demonstrated that the insulin sensitivity was clinically augmented by the continuous infusion of active GLP-1 peptide [10]. Regarding the GLP-1 analog treatment, lack of consistent and convincing evidence of improved insulin sensitivity by the exenatide treatment has provoked confusing discussion about the clinical effects of GLP-1 analog on insulin sensitivity [11–13]. As regards hospitalized diet-therapy and the GLP-1 analog treatment, there is no clinical study that demonstrates the effective changes in insulin sensitivity assessed by the hyperinsulinemic-euglycemic clamp method. Furthermore, the short-term effects of introducing liraglutide-therapy on insulin sensitivity have not yet been clinically clarified in patients with type 2 DM.

We investigated whether liraglutide, a GLP-1 analog, could enhance insulin sensitivity as assessed by the hyperinsulinemic-euglycemic clamp in patients with uncontrolled type 2 DM.

## 2. Materials and Methods

### 2.1. Study Population and Protocol

We prospectively recruited stable but uncontrolled type 2 diabetes patients (hemoglobin A1c; HbA1c > 7.0%) at the outpatient clinic of the Diabetes Care Center in Jinnouchi Hospital between March 2011 and June 2014. The patients who agreed to get treatment in hospital for diabetes were admitted to Jinnouchi Hospital and treated for 4 weeks. Exclusion criteria were type 1 DM, age > 80 years, unstable cardiovascular diseases, active inflammation, severe liver diseases, dementia, chronic kidney disease stage ≥4, disability in bed, and cancer. Patients with newly diagnosed diabetes without any treatments and patients treated with thiazolidinediones were also excluded. For lifestyle modification, all patients were instructed for dietary-therapy by nutritionists and exercise-therapy by exercise trainers. Before starting the hospitalized intensive insulin- or liraglutide-therapies, fasting and postprandial blood samples were collected from the antecubital vein in the morning and the hyperinsulinemic-euglycemic clamp was performed. On admission, the ongoing oral medications for diabetes treatments (sulfonylurea, alpha-glucosidase inhibitors, and glinides) were adjusted and dipeptidyl peptidase-4 inhibitors were stopped but metformin was continued with the same doses as before admission. At the same time, all patients nonrandomly started with treatments of either gradually increasing doses of liraglutide (Novo Nordisk, liraglutide; 0.3 mg/day for 1 week and then 0.6 mg/day for 1 week, followed by 0.9 mg/day [maximum clinical dose in Japan] for 2 weeks) or multiple daily subcutaneous insulin injections (intensive insulin-therapy). The intensive insulin-therapy group was treated with insulin aspart (Novo Nordisk), insulin lispro (Eli Lilly), or insulin glulisine (Sanofi Aventis) before each meal and insulin degludec (Novo Nordisk) or insulin glargine (Sanofi Aventis) at bedtime. During the hospitalization therapies, frequent adjustments were made to the intensive insulin regimens to achieve fasting plasma glucose (FPG) of approximately 100 mg/dL and a postprandial glucose of <180 mg/dL. After the 4-week hospitalized treatment, fasting and postprandial blood samples and a second hyperinsulinemic-euglycemic clamp were performed.

This study was conducted in accordance with the Declaration of Helsinki. The study protocol was approved by the Human Ethics Review Committee of Jinnouchi Hospital, and a signed consent form was obtained from each patient.

### 2.2. Hospitalized Lifestyle Modification by Diet-Therapy and Exercise Instruction

The hospitalized lifestyle modification therapy was provided by physicians, nutritionists, nurses, and exercise trainers on admission [14]. All patients were provided with printed information regarding diet and exercise to treat DM. The dietary-therapy was taught to each patient in the first week and managed hospital meals (total calories, ideal body weight × 26–28 kcal, carbohydrate 40%, protein 1.2 g/kg, lipids ≤300 mg/day cholesterol, nonsaturated and saturated fatty acids <2.0, no alcohol, and NaCl 6-7 g/day) were provided to all patients. Exercise instruction was provided with the use of treadmills and bicycle ergometers in the hospital exercise room and by walking around the hospital. Exercise trainers gave instructions regarding moderate-intensity physical exercise during each 30-minute session. The exercise intensity was determined by the Borg scale (Borg scale: 11 to 13). At least 30 minutes of daily exercise was recommended.

### 2.3. Blood Sampling and Measurement of Clinical Parameters

Fasting and postprandial blood samples were collected from the antecubital vein in the morning before starting the hospitalized therapies and after the 4 weeks of treatment with liraglutide or insulin. Blood analyses were conducted in the hospital laboratory for the measurement of blood glucose, HbA1c, insulin, and C-peptide immunoreactivity (CPR).

### 2.4. Hyperinsulinemic-Euglycemic Clamp Examination

**Figure 1:**
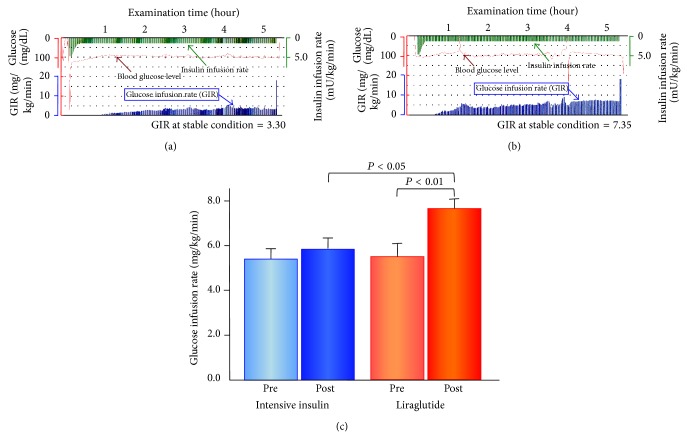
Glucose infusion rate in euglycemic-hyperinsulinemic clamp before and after the 4-week intensive insulin-therapy and liraglutide-therapy. (a) and (b) Representative records of measurement of glucose infusion rate during euglycemic-hyperinsulinemic clamp examination before and after the 4-week liraglutide-therapy. Real trace recordings demonstrate representative results of euglycemic-hyperinsulinemic clamp in a 77-year-old male with type 2 DM. He was treated by sulfonylurea before the introduction to liraglutide-therapy. His GIR value was increased from 3.30 (a) to 7.35 (b). (c) Actual measured values of the glucose infusion rate in euglycemic-hyperinsulinemic clamp before and after the 4-week intensive insulin-therapy and liraglutide-therapy. Bar graphs represent the actual measured values of the glucose infusion rate (GIR) of the mean and standard error of mean in patients with intensive insulin-therapy (*n* = 15) and liraglutide-therapy (*n* = 16). The intergroup comparisons of the posttreatment GIR values in liraglutide-therapy were significantly higher than those in intensive insulin-therapy (unpaired *t*-test, *P* < 0.05). The intragroup comparisons of the posttreatment GIR values were significantly higher than the pretreatment values in liraglutide-therapy (paired *t*-test, *P* < 0.01). Pre: pretreatment; Post: posttreatment.

At hospital admission before starting the therapies and at 4 weeks following each treatment, insulin sensitivity was evaluated by a hyperinsulinemic-euglycemic clamp examination using an artificial pancreas (Nikkiso STG-22 or STG-55; Nikkiso, Tokyo, Japan, Figures 1(a) and 1(b)) [15]. Daily medications were withheld in the morning of the clamp procedure. Insulin was given as intravenous loading doses (starting from 4.77 mU/kg/min and were gradually decreased to 1.67 mU/kg/min; plasma insulin concentration was 100 mU/L) over 10 minutes followed by a continuous infusion at 1.5 mU/kg/min for 120 minutes. Plasma glucose concentrations were maintained at 100 mg/dL by a variable infusion of 10% glucose. The stable glucose infusion rate (GIR: mg/kg/min) was calculated as the index of insulin sensitivity (Figures 1(a) and 1(b)) [14].

### 2.5. Statistical Analysis

Based on results of previous studies [10, 16] and our preliminary examination in our hospital, power analysis indicated that an enrolment of 28 patients was required to detect a mean increase in GIR of 1.5 ± 1.5 mg/kg/min in the liraglutide group and 0.1 ± 1.0 mg/kg/min in the intensive insulin group, with a power of 80% and a two-sided alpha of 0.05.

The results of the normally distributed continuous variables (determined by the Shapiro-Wilk test) were expressed as the mean (standard deviations; SD), while those of the continuous variables with skewed distributions were expressed as median values (interquartile ranges). Differences in the baseline characteristics of the two groups were analyzed by Student's* t*-test, the Mann-Whitney *U* test, or Fisher's exact test for categorical data, as appropriate. Either paired Student's* t*-test or Wilcoxon test was used to analyze the effect of each treatment. The differences between treatment groups in the changes in GIR from baseline to week 4 were also assessed using analysis of covariance (ANCOVA) with adjustment for the baseline measure of body mass index (BMI) and HbA1c. Logistic regression analysis was used to evaluate the association between the increased insulin sensitivity (GIR increase > 0.5 mg/kg/min) and the baseline clinical variables including liraglutide-therapy allocation. Associations between groups and all other parameters were analyzed by univariate logistic regression analysis followed by multivariate logistic regression analysis with the forced inclusion model, and the Hosmer-Lemeshow goodness-of-fit statistic was calculated. To determine the relation between changes in various clinical parameters and the changes in GIR as the insulin-sensitivity measure in the liraglutide group, correlations between variables of interest were analyzed using Pearson's correlation coefficient. A *P* value <0.05 was considered statistically significant. Statistical analyses were performed using the Statistical Package for Social Sciences version 19 (SPSS Inc., IBM, Tokyo, Japan) and SAS version 9.4 (SAS Institute Inc., Cary, NC, USA).

## 3. Results

### 3.1. Baseline Clinical Characteristics

The present study consisted of 31 stable Japanese patients with uncontrolled type 2 DM. The clinical baseline characteristics of the patients and of each group are shown in Table 1. The mean age was 61.1 years, 71.0% were male, mean BMI was 26.6 kg/m^2^, HbA1c was 8.8%, and FPG was 151.6 mg/dL. At enrollment, 29.7% were treated with insulin. The baseline characteristics of patients in the intensive insulin group were similar to those of the liraglutide group. The levels of BMI, HbA1c, FPG, postprandial plasma glucose (PPPG), duration of diabetes, and fasting blood CPR were not significantly different between the groups. There was no significant difference in the insulin sensitivity assessed by GIR values in the hyperinsulinemic-euglycemic clamp examination between the groups at baseline (Table 2).

### 3.2. Changes in Glucose Metabolic Parameters before and after the Therapies

At the end of the hospitalized therapies, 21.4 ± 9.6 units/day of insulin was used for the intensive insulin-therapy and 0.9 mg/day of liraglutide was administered to all the allocated patients. Using the hospitalized multidisciplinary approach including the appropriate dietary- and exercise-therapies, all patients of both treatment groups presented with significant improvements in HbA1c (mean ± SD; pretreatment 8.8 ± 1.0% to posttreatment 7.9 ± 0.8%, *P* < 0.001), FPG (151.0 ± 31.6 to 123.5 ± 35.1 mg/dL, *P* < 0.01), PPPG (251.2 ± 64.2 to 153.1 ± 45.5 mg/dL, *P* < 0.001), and BMI (26.6 ± 2.7 to 25.6 ± 2.5, *P* < 0.001). As shown in Table 2, HbA1c, PPPG, and BMI were significantly decreased in both groups, but decreases in HbA1c, FPG, and PPPG were significantly greater in the intensive insulin group than in the liraglutide group. The fasting and postprandial CPR values and C-peptide index levels were significantly increased and the changes for both were significantly greater in the liraglutide group compared with the intensive insulin group (Table 2).

### 3.3. Changes in Insulin Sensitivity Assessed by Glucose Clamp before and after Therapies

**Figure 2:**
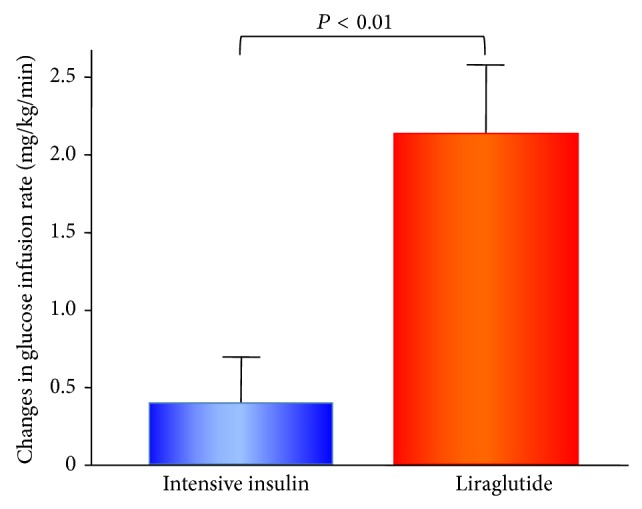
Changes in glucose infusion rate in euglycemic-hyperinsulinemic clamp after the 4-week intensive insulin-therapy and liraglutide-therapy. Bar graphs represent the amount of change in glucose infusion rate (GIR) as the mean and standard error of mean in patients with intensive insulin-therapy (*n* = 15) and liraglutide-therapy (*n* = 16). Change in GIR (mg/kg/min) = (GIR after 4 weeks of therapy) − (GIR at admission). The intergroup comparisons of the changes in GIR values in liraglutide-therapy were significantly higher than those in intensive insulin-therapy (unpaired *t*-test, *P* < 0.01).

During the 4-week short-term hospitalized treatments with controlled lifestyle management, the liraglutide-therapy, but not the intensive insulin-therapy, exhibited a significant increase in the systemic insulin sensitivity assessed by GIR (Table 2 and Figure 1). Both the insulin- and liraglutide-therapy increased GIR during each therapy, while the GIR values at the end of the hospitalized therapies were significantly greater in the liraglutide group than in the intensive insulin group (*P* < 0.05, Figure 1). The changes in GIR were also significantly larger in the liraglutide group than in the intensive insulin group (*P* < 0.01, Table 2 and Figure 2).

In the ANCOVA model using the baseline BMI and HbA1c levels as covariates, the liraglutide-treatment demonstrated significantly greater change in GIR compared with the intensive insulin-therapy (*P* < 0.05), indicating that the significant association between the liraglutide-therapy and the increased insulin sensitivity was independent of the baseline BMI and HbA1c levels in patients with uncontrolled type 2 DM.

As shown in Table 3, the univariate logistic regression analysis demonstrated that the baseline BMI and the liraglutide-therapy allocation were significantly correlated with the increased insulin sensitivity defined as the changes in GIR >0.5 mg/kg/min. The forced inclusion multivariate logistic regression analysis with BMI and liraglutide-therapy revealed that only liraglutide-therapy was significantly correlated with the increased insulin sensitivity (odds ratio 5.076, 95% confidence interval 1.204 to 25.0, and *P* = 0.047). The Hosmer-Lemeshow statistic was appropriate (*P* = 0.19). Because weight loss effects of liraglutide are well known, we included changes in BMI in the univariate logistic regression analysis and found that reduction in BMI was not significantly associated with improved GIR (odds ratio 2.22, 95% confidence interval 0.69 to 7.14, and *P* = 0.18) in the whole population. In the multivariate logistic regression analysis with the forced inclusion model for reduction in BMI and therapies, liraglutide-therapy was still significantly associated with improved GIR (odds ratio 6.25, 95% confidence interval 1.14 to 7.69, and *P* = 0.04). The Hosmer-Lemeshow statistic was appropriate (*P* = 0.14).

### 3.4. Correlation between Changes in Clinical Parameters and Changes in Insulin Sensitivity before and after the Liraglutide-Therapies

To determine the associated factors between the liraglutide-induced insulin sensitivity and the changes in clinical parameters, we investigated the correlation coefficient between GIR increase and the changes in variables during the liraglutide-therapy. As shown in Table 4, the changes in GIR are not significantly correlated with the changes in BMI, HbA1c, and FPG. Furthermore, the changes in GIR did not have any correlation with the changes in the other clinical parameters during the liraglutide-therapy.

## 4. Discussion

We demonstrated that the 4-week short-term liraglutide-therapy enhanced insulin sensitivity in patients with uncontrolled type 2 DM attending a clinical practice. The treatment-related decrease in BMI by liraglutide was not significantly correlated with the increase in insulin sensitivity.

Impaired insulin sensitivity and increased insulin resistance are profoundly involved in the pathogenesis and pathophysiology of type 2 DM and its associated cardiovascular complications [17]. Therapies for improving or enhancing insulin sensitivity will hopefully bring beneficial clinical effects on the metabolic disorders and lifestyle-related diseases including obesity and diabetes [18]. Recently, clinical attention has been focused on the incretin-related medicines such as GLP-1 analog as a new treatment strategy for diabetes and obesity [5, 6]. It has been suggested that GLP-1 analog may have the ability of enhancing insulin sensitivity [7], but some studies have demonstrated controversial clinical results regarding the augmentation of insulin sensitivity by GLP-1 analog [11–13]. In the present study, we investigated the change in insulin sensitivity as assessed by the hyperinsulinemic-euglycemic clamp. This study clearly demonstrated that the 4-week short-term comprehensive intervention with liraglutide and lifestyle management could provide significant improvement of insulin sensitivity in uncontrolled diabetes patients. The short-term liraglutide-therapy has effectively been shown to reduce BMI and HbA1c [19], but the changes in GIR were not significantly correlated with the changes in BMI and HbA1c during the treatment and there was no effect of the baseline BMI on the GIR changes owing to liraglutide. These results suggest that liraglutide-induced insulin sensitivity augmentation could potentially be beneficial for the treatment of a wide spectrum of uncontrolled type 2 diabetes patients. Furthermore, with regard to disease mechanisms, coadministration of liraglutide and insulin secretion stimulators or insulin products could be reasonable and effective medical therapies for type 2 DM.

Previously, studies have shown that metformin and thiazolidinedione can clinically be effective in improving insulin sensitivity [8]. In the present study, the magnitude of increase in insulin sensitivity by liraglutide (GIR increased by 63%) was greater than the previously reported values of metformin (GIR increased by 18%) [8] or pioglitazone (GIR increased by 36%) [8]. We consider that the improvement of insulin sensitivity by liraglutide would be useful and beneficial in investigating the fundamental pathophysiology of diabetes. Because we excluded patients pretreated with thiazolidinediones, it was not possible to assess any additional positive effect of liraglutide on insulin sensitivity in patients pretreated with thiazolidinediones. Further studies are needed to investigate the synergistic effect of thiazolidinediones and liraglutide on insulin sensitivity.

In patients with uncontrolled diabetes, the use of glucose-lowering therapies associated with relieving high glucose-mediated glucotoxicity might potentially bring amelioration of insulin resistance [20]. The present results demonstrated that hospitalized lifestyle management with adequate diet, exercise, and intensive insulin-therapies provided significantly greater improvement in the diabetic condition as observed with the decrease in levels of HbA1c and the fasting and postprandial blood glucose compared with the liraglutide-therapy. However, the reduced glucotoxicity by intensive insulin-therapy failed to show significantly improved effects on insulin resistance. Intensive insulin-therapy with adequate dietary- and exercise-therapies significantly decreased BMI but insulin sensitivity was not significantly improved. Conversely, liraglutide-therapy significantly improved insulin sensitivity under the same conditions. These results indicate that the short-term relieving of glucotoxicity alone is not enough to achieve the significant improvement of insulin sensitivity in uncontrolled diabetes patients, and the different clinical effects of each antidiabetic medicine should be carefully considered in the practical situation.

Cellular and molecular mechanisms of the liraglutide-induced insulin sensitivity augmentation are still under investigation. Chen et al. [21] reported that GLP-1 analog amplifies insulin signaling in adipocytes by upregulating insulin signaling molecules including basal expression of insulin receptor, insulin receptor substrate-1, and glucose transporter-4. Liraglutide has been shown to ameliorate insulin resistance through upregulation of glucose transporter-4 [21]. AMPK activation in muscle and liver induces insulin sensitivity augmentation and provides favorable effects on glucose metabolism [9]. Previous studies demonstrated that liraglutide successfully activated AMPK [8] and the liraglutide-AMPK cascade might potentially lead to increasing insulin sensitivity [9]. Liraglutide has been shown to decrease oxidative stress [22, 23] and inflammation [8, 24] and the suppression of oxidative stress and inflammation might link to the amelioration of insulin resistance. Liraglutide has been shown to improve endothelial [25] and cardiac function [26], all of which might result in increasing systemic insulin sensitivity. In addition, it has been reported that liraglutide could reduce endogenous glucose release and glucagon secretion [27, 28], which might have contributed to the changes in GIR in the present study. Taken together, these effects of liraglutide can exhibit improvement of insulin sensitivity through its unique extrapancreatic effects.

The present study has several limitations. It included a relatively small number of patients and the study protocol was prospective in design and not a randomized clinical trial. Patients with larger BMI tended to be treated by liraglutide and this selection bias might have affected the results. However, the multivariate logistic regression analysis and ANCOVA demonstrated the independent significance of clinically based liraglutide-therapy on the increased insulin sensitivity. Thus, we believe that liraglutide could in part exhibit beneficial effects on insulin sensitivity in uncontrolled type 2 DM patients. Another limitation of the study was that assessment of hospitalized lifestyle intervention could not have been performed in a quantitative manner. Because the present results were achieved by hospitalized short-term intervention, it is necessary to investigate and confirm the long-term and chronic effects of liraglutide on insulin sensitivity.

## 5. Conclusions

In this study, the short-term use of liraglutide enhanced insulin sensitivity in patients with type 2 diabetes. Liraglutide could exhibit favorable effects on the pathogenesis of metabolic disorders in patients with type 2 DM by increasing insulin sensitivity as an extrapancreatic action.

## Figures and Tables

**Table 1:** Baseline clinical characteristics.

	Total patients (*n* = 31)	Liraglutide (*n* = 16)	Intensive insulin (*n* = 15)	*P* value

Age (years)	61.1 ± 10.5	62.3 ± 12.8	59.8 ± 7.7	*P* = 0.53
Sex (male)	71.0%	81.3%	60.0%	*P* = 0.19
Body mass index (kg/m^2^)	26.6 ± 2.7	27.3 ± 2.7	25.8 ± 2.5	*P* = 0.12
Hypertension	77.4%	75.0%	80.0%	*P* = 0.74
Dyslipidemia	58.1%	62.5%	53.3%	*P* = 0.61
Current smoking	19.4%	12.5%	26.7%	*P* = 0.39
Duration of diabetes (years)	11.1 ± 9.4	11.8 ± 9.2	10.3 ± 9.9	*P* = 0.67
Family history of diabetes	58.6%	43.8%	73.3%	*P* = 0.15
Hemoglobin A1c (%)	8.8 ± 1.0	8.7 ± 1.0	8.9 ± 0.9	*P* = 0.66
Fasting plasma glucose (mg/dL)	151.6 ± 31.7	149.8 ± 33.3	153.6 ± 30.9	*P* = 0.74
Fasting blood insulin (*µ*U/mL)	8.5 (4.5–14.3)	9.0 (4.8–17.0)	5.2 (4.1–12.5)	*P* = 0.17
Fasting blood CPR (ng/mL)	2.2 ± 1.1	2.4 ± 1.2	2.0 ± 0.9	*P* = 0.35
Antidiabetic medicines	—	—	—	—
Sulfonylureas	28.5%	25.0%	26.0%	*P* = 0.92
Metformin	12.9%	12.5%	13.3%	*P* = 1.00
Alpha-glucosidase inhibitor	6.5%	6.3%	6.7%	*P* = 1.00
DPP-4 inhibitor	16.1%	12.5%	20.0%	*P* = 0.65
Insulin	35.5%	43.8%	26.7%	*P* = 0.46
Insulin use (units/day)	29.7 ± 18.2	33.9 ± 20.6	23.8 ± 14.3	*P* = 0.34

CPR: C-peptide radioimmunoreactivity; DPP-4: dipeptidyl peptidase.

**Table 2:** Changes in body weight and glucose metabolic parameters before and after therapies.

	Liraglutide (*n* = 16)		*P* value	Intensive insulin (*n* = 15)		*P* value
	Baseline	4 weeks		Baseline	4 weeks	
Body mass index	27.3 ± 2.7	26.1 ± 2.3	*P* < 0.001	25.8 ± 2.5	25.1 ± 2.6	*P* = 0.003
Absolute change	−1.3 ± 0.6			−1.0 ± 0.8		
% change (%)	−4.3 ± 2.4			−3.2 ± 2.1		
HbA1c (%)	8.7 ± 1.0	8.0 ± 0.8	*P* < 0.001	8.9 ± 0.9	7.7 ± 0.8	*P* < 0.001
Absolute change (%)	−0.6 ± 0.4^‡^			−1.2 ± 0.9^‡^		
% change (%)	−7.0 ± 4.3^‡^			−12.8 ± 9.9^‡^		
Fasting plasma glucose (mg/dL)	149.8 ± 33.3	138.0 ± 41.3	*P* = 0.38	152.3 ± 30.7	108.1 ± 17.9	*P* < 0.001
Absolute change	−11.8 ± 51.5^‡^			−47.4 ± 36.5^‡^		
% change (%)	−3.4 ± 34.4^‡^			−28.2 ± 17.5^‡^		
Fasting blood CPR (ng/mL)	2.4 ± 1.2	3.7 ± 1.3	*P* = 0.004	1.8 ± 0.7	1.4 ± 0.5	*P* = 0.004
Absolute change	1.3 ± 1.5^†^			−0.4 ± 0.4^†^		
% change (%)	84.2 ± 92.6^†^			−17.2 ± 25.3^†^		
C-peptide index	1.65 ± 0.74	2.84 ± 1.09	*P* < 0.001	1.26 ± 0.51	1.28 ± 0.42	*P* = 0.77
Absolute change	1.19 ± 1.01^†^			0.02 ± 0.27^†^		
% change (%)	89.7 ± 65.1^†^			11.4 ± 34.5^†^		
Postprandial plasma glucose (mg/dL)	242.4 ± 62.2	166.9 ± 47.0	*P* < 0.001	262.9 ± 67.8	134.7 ± 37.6	*P* < 0.001
Absolute change	−75.6 ± 58.6^‡^			−128.3 ± 59.6^‡^		
% change (%)	−28.2 ± 19.6^†^			−47.0 ± 13.8^†^		
Postprandial blood CPR (ng/mL)	5.04 ± 3.40	6.73 ± 2.03	*P* = 0.03	3.93 ± 2.09	3.90 ± 1.50	*P* = 0.95
Absolute change	1.45 ± 2.70			−0.27 ± 1.45		
% change (%)	62.2 ± 80.3^‡^			9.9 ± 37.4^‡^		
GIR (mg/kg/min)	5.51 ± 2.33	7.64 ± 1.81^‡^	*P* < 0.001	5.46 ± 2.21	5.86 ± 2.51^‡^	*P* = 0.27
Absolute change	2.13 ± 1.77^†^			0.40 ± 1.34^†^		
% change (%)	63.3 ± 75.2^‡^			11.8 ± 31.5^‡^		

HbA1c, hemoglobin A1c; CPR, C-peptide radioimmunoreactivity; ^†^ *P* < 0.01, and ^‡^ *P* < 0.05 liraglutide-therapy versus intensive insulin-therapy.

**Table 3:** Logistic regression analysis for the improvement of GIR (>0.5 mg/min/kg) in patients with uncontrolled type 2 DM.

Baseline variable	Univariate regression			Multivariate regression using forced inclusion model		
	OR	95% CI	*P*	OR	95% CI	*P*

Age (per year)	0.997	0.932 to 1.068	0.94		—	
Gender (male)	1.805	0.377 to 8.621	0.46		—	
Body mass index (per 1.0)	1.241	0.925 to 1.665	0.15	1.161	0.847 to 1.591	0.35
Hypertension (yes)	4.167	0.664 to 26.31	0.128		—	
Dyslipidemia (yes)	1.835	0.432 to 7.752	0.411		—	
Current smoker (yes)	0.333	0.051 to 2.179	0.251		—	
Hemoglobin A1c (per 0.1; %)	1.147	0.536 to 2.455	0.724		—	
Fasting plasma glucose (per 1.0; mg/dL)	0.996	0.974 to 1.019	0.752		—	
Fasting blood CPR (per 1.0; ng/mL)	1.265	0.615 to 2.601	0.523		—	
Duration of diabetes (per 1.0; years)	0.981	0.908 to 1.059	0.623		—	
Liraglutide-therapy (yes)	5.989	1.231 to 28.57	0.024	5.076	1.024 to 25.00	0.047

OR, odds ratio; CI, confidence interval; CPR, C-peptide radioimmunoreactivity.

Hosmer-Lemeshow *P* = 0.19 in multivariate analysis.

**Table 4:** Correlation between changes in glucose infusion rate at euglycemic-hyperinsulinemic clamp and clinical variables in patients treated with liraglutide.

	*r*	*P* value
Changes in body mass index	−0.086	0.760
Changes in hemoglobin A1c (%)	−0.101	0.710
Changes in fasting blood glucose (mg/dL)	−0.165	0.540
Changes in fasting blood insulin (*µ*U/mL)	0.128	0.650
Changes in fasting blood CPR (ng/mL)	−0.154	0.583
Changes in postprandial blood glucose (mg/dL)	−0.383	0.143
Changes in postprandial blood insulin (*µ*U/mL)	0.067	0.819
Changes in postprandial blood CPR (ng/mL)	−0.293	0.309

CPR: C-peptide radioimmunoreactivity.
